# Contribution of Imaging in the Diagnosis of Cholangiocarcinoma in Choledochal Cyst

**DOI:** 10.1155/2018/8346232

**Published:** 2018-12-12

**Authors:** Djivèdé Witchékpo Maurice Mohamed Akanni, Kouassi Paul N'zi, Kofi-Mensa Savi de Tové, Laura Petrovai

**Affiliations:** ^1^Faculté de Médecine, Université de Parakou, Benin; ^2^Centre Hospitalier de Dunkerque, France; ^3^Institut de Cardiologie d'Abidjan, Université Félix Houphouët-Boigny, Côte d'Ivoire

## Abstract

The choledochal cyst is a rare congenital malformation of the bile ducts. It is considered as a precancerous state. The incidence of cancer in choledochal cyst increases with age and occurs around 32 years old. Therefore, young adults are often involved. In adults, clinical signs are rare and unspecific. We are reporting here the case of a cholangiocarcinoma in choledochal cyst in young adult diagnosed by ultrasonography, CT scan, and MRI and confirmed by histopathological examination.

## 1. Introduction

The cystic dilatation of the common bile duct is a rare congenital malformation of the bile ducts [1]. It is considered as a precancerous state [1]. The incidence of cancer on the choledochal cyst increases with age and the average age for a cancer diagnosis is 32 years old [1].

We are reporting here a case of a suspected cholangiocarcinoma in choledochal cyst by medical imaging and confirmed by anatomopathological examination.

## 2. Case Presentation

In January 2012, a 38-year-old Caucasian woman went to the Emergency Room of the Dunkerque Hospital Center (France), for epigastric pain, no fever, along with jaundice which had been evolving for about a week. Her antecedents were appendectomy, tonsillectomy, and scoliosis with Carrington's stems. The clinical examination noted a swelling of the epigastrium and the right hypochondrium and, above all, a palpable mass was localized at the right hypochondrium. Biochemical analysis showed a liver cytolysis (transaminases: 550IU/L, 10 times greater than the normal threshold), a cholestasis (total bilirubin: 25mg/dl, direct bilirubin: 18mg/dl, indirect bilirubin: 7mg/dl), and an increase in gamma glutamyl transpeptidase: 467IU/L. Lipase and pancreatic amylase were normal.

**Figure 1:**
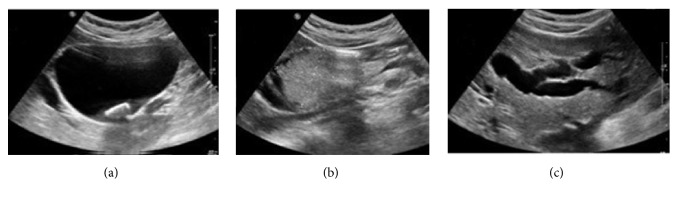
Ultrasound transverse sections. (a) Cystic mass with a gallstone independent of the gallbladder. (b) Intracystic tissue bud. (c) Dilatation of the intrahepatic bile ducts.

**Figure 2:**
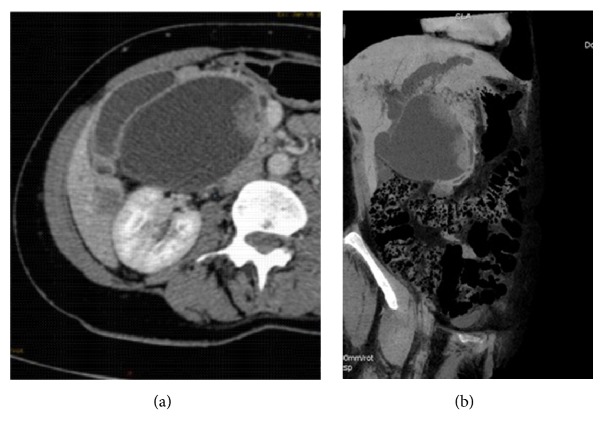
CT sections after injection of iodized product at portal venous phase. (a) Axial section: cystic mass with secondary lesion of the VI segment. (b) Oblique reconstruction showing mass relationship with the bile ducts.

**Figure 3:**
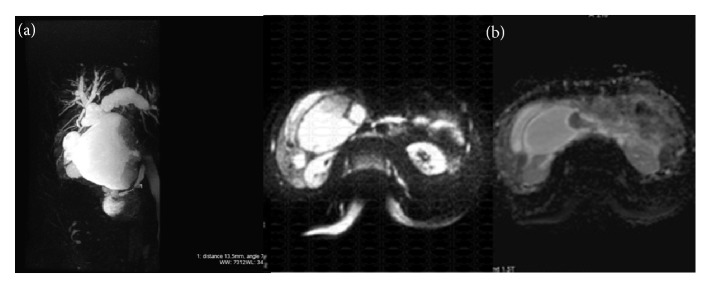
Biliary MRI. (a) Front MIP reconstruction of the bile ducts and cystic mass. (b) The diffusion sequence showed high signal intensity of intracystic bud and the apparent diffusion coefficient (ADC) map shows low signal intensity of lesions characteristic of tumor lesion.

An abdominal ultrasound was performed and revealed a cystic mass measuring 15 cm x 10 cm, independent of the gallbladder, located between the portal vein confluence and the duodenum. Intracystic gallstone and an intraluminal tissue bud were present at the cystic wall. There was no color flow on Doppler exam in the mass. In addition, there was intrahepatic bile duct dilation, particularly noticeable on the left (Figure 1). An abdominal-pelvic CT scan was immediately performed, along with an injection of iodinated contrast medium. It showed at portal venous phase the well-defined cystic mass independent of the gallbladder, extended between the confluence of the portal vein and the duodenum, and the intracystic bud that enhances after injection. CT-scan also revealed centimetric hypodense nodules not enhanced after contrast injection in segment VI of the liver and dilation of the left intrahepatic bile ducts. The oblique reconstruction showed the relationship of this cystic mass with the biliary tree (Figure 2). Biliary MRI indicated the relation between the cystic mass and the bile ducts. The diffusion sequence performed during this examination confirmed the malignancy of the liver's segment VI nodules and the intracystic tissue bud (Figure 3). The diagnosis of cholangiocarcinoma in a choledochal cyst with hepatic metastases was retained. Transferred to the University Hospital of Lille, the patient underwent a resection of the cystic mass, a cholecystectomy, and a biliodigestive anastomosis.

**Figure 4:**
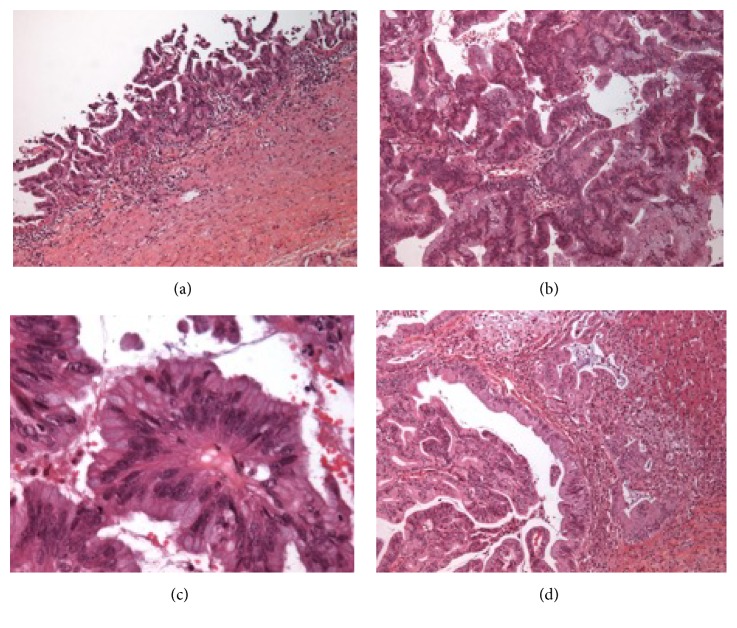
Histopathology. (a) Endocytic adenocarcinoma x10. (b) Adenocarcinoma bud x10. (c) Adenocarcinoma bud x40. (d) Degeneration of the liver x10.

Histopathological examination of the intracystic tissue bud and of the liver's nodules of segment VI showed an appearance of intraductal and infiltrating adenocarcinoma (Figure 4). During the patient's hospitalization, GEMZAR CYSPLATINE-type chemotherapy was set up after the multidisciplinary consultation meeting.

## 3. Discussion

The choledochal cyst is a rare disease in Europe and Africa but more common in Asia.

33 to 50% of the cases in the world are reported in Japanese literature [2]. It is three time prevalent in female than male [3]. Most of choledochal cysts are diagnosed during childhood before the age of 10, but 20% of choledochal cyst are diagnosed during adulthood [1] as in our study.

Choledochal cyst is a precancerous condition with cancer occurring more often and earlier for these patients [2, 3]. The tumor occurs mainly in young adults whose anomaly was not known early and/or who have undergone choledochal cyst surgical treatment during their childhood. Todani et al. made a distinction between primary cancers on choledochal cyst, developed in patients with no surgical treatment, and secondary cancers developed in patients who previously had choledochal cyst surgery [2, 3]. In our case, the patient had not got choledochal cyst surgical treatment and therefore had a primary cancer.

The histological types of cancer observed in patients with choledochal cyst are as follows: adenocarcinoma (73% to 84% of cases), anaplastic carcinoma (10% of cases), undifferentiated carcinoma (5% to 7% of cases), squamous cell carcinoma (5% of cases), and other cancers (1.5% of cases) [2, 4]. The patient of our study had adenocarcinoma.

The classical triad of vague intermittent upper abdominal pain, intermittent jaundice, and right upper abdominal mass is rarely seen [5]. Jaundice is seen more frequently with infants [5]. In children, the signs are intermittent then progressive [5]. In adults, however, the diagnosis is sometimes delayed due to the scarcity and the nonspecificity of the clinical signs. Finally, some of the choledochal cysts are revealed by an inaugural complication such as cancer [5–7] as in our case study. Hence lies the importance of medical imaging for the anatomical assessment of the bile ducts when suspecting the involvement of the biliary tree [8].

The first scanning method of the biliary tree is an abdominal ultrasound. It is a noninvasive examination accessible quickly and inexpensively [8]. The common bile duct is almost always visible. When the size of the choledochal cyst is very large, ultrasonography does not allow the analysis of the distal part of the choledochal duct and the surrounding anatomical structures [8]. In our case, the size of the choledochal cyst as well as the intracystic tumor bud justified the use of CT and MRI. CT and MRI are indicated to assess the lesion and to study the relations with the neighboring structures, especially when one suspects a malignant transformation of the choledochal cyst [8, 9]. These two medical imaging methods helped us to rule out the possibility of a low bilious obstruction such as gallstone, pancreatic head tumor, and duodenal tumor. In addition, the CT scan and the MRI diffusion sequence also made it possible to diagnose liver metastases. The combination of these radiological investigations made it possible to suspect an adenocarcinoma in choledochal cyst of our patient. This diagnosis was confirmed by the histopathological examination.

## 4. Conclusion

This case of cholangiocarcinoma in choledochal cyst showed the importance and necessity of combining several imaging modalities (ultrasonography, CT scan, and MRI) to reach diagnosis. Early abdominal ultrasonography is recommended in case of intermittent abdominal pain and cholestasis in young adults, in order not to miss diagnosis of choledochal cyst.
